# Identification and Validation of a Six Immune-Related Genes Signature for Predicting Prognosis in Patients With Stage II Colorectal Cancer

**DOI:** 10.3389/fgene.2021.666003

**Published:** 2021-05-04

**Authors:** Xianzhe Li, Minghao Xie, Shi Yin, Zhizhong Xiong, Chaobin Mao, Fengxiang Zhang, Huaxian Chen, Longyang Jin, Ping Lan, Lei Lian

**Affiliations:** ^1^Department of Colorectal Surgery, The Sixth Affiliated Hospital of Sun Yat-sen University, Guangzhou, China; ^2^Guangdong Provincial Key Laboratory of Colorectal and Pelvic Floor Diseases, Guangdong Institute of Gastroenterology, The Sixth Affiliated Hospital of Sun Yat-sen University, Guangzhou, China

**Keywords:** colorectal cancer, immune-related genes, prognosis, stage II, tumor immune microenvironment

## Abstract

**Background:**

Immune-related genes (IRGs) play important roles in the tumor immune microenvironment and can affect the prognosis of cancer. This study aimed to construct a novel IRG signature for prognostic evaluation of stage II colorectal cancer (CRC).

**Methods:**

Gene expression profiles and clinical data for stage II CRC patients were collected from the Cancer Genome Atlas and Gene Expression Omnibus database. Univariate, multivariate Cox regression, and least absolute shrinkage and selection operator regression were used to develop the IRG signature, namely IRGCRCII. A nomogram was constructed, and the “Cell Type Identification by Estimating Relative Subsets of RNA Transcripts” (CIBERSORT) method was used to estimate immune cell infiltration. The expression levels of genes and proteins were validated by qRT-PCR and immunohistochemistry in 30 pairs of primary stage II CRC and matched normal tissues.

**Results:**

A total of 466 patients with stage II CRC were included, and 274 differentially expressed IRGs were identified. Six differentially expressed IRGs were detected and used to construct the IRGCRCII signature, which could significantly stratify patients into high-risk and low-risk groups in terms of disease-free survival in three cohorts: training, test, and external validation (GSE39582). Receiver operating characteristics analysis revealed that the area under the curves of the IRGCRCII signature were significantly greater than those of the OncotypeDX colon signature at 1 (0.759 vs. 0.623), 3 (0.875 vs. 0.629), and 5 years (0.906 vs. 0.698) disease-free survival, respectively. The nomogram performed well in the concordance index (0.779) and calibration curves. The high-risk group had a significantly higher percentage of infiltrated immune cells (e.g., M2 macrophages, plasma cells, resting mast cells) than the low-risk group. Finally, the results of qRT-PCR and immunohistochemistry experiments performed on 30 pairs of clinical specimens were consistent with bioinformatics analysis.

**Conclusion:**

This study developed and validated a novel immune prognostic signature based on six differentially expressed IRGs for predicting disease-free survival and immune status in patients with stage II CRC, which may reflect immune dysregulation in the tumor immune microenvironment.

## Introduction

Colorectal cancer (CRC) is the third most common cancer and a leading cause of cancer-related mortality worldwide (Fitzmaurice et al., 2015; Bray et al., 2018). Stage II CRC involves a local tumor without lymph node metastasis and accounts for approximately 25% of all CRC cases (Quah et al., 2008; Lee and Chu, 2017). Surgical operation is the main stay treatment for stage II CRC, but approximately 15–25% of patients still develop relapse or death within 5 years after surgery (Hari et al., 2013). While post-operative adjuvant chemotherapy is now the standard treatment for stage III CRC, the benefit of chemotherapy in stage II CRC remains controversial (Schippinger et al., 2007; Glynne-Jones et al., 2017; Fotheringham et al., 2019). Therefore, reliable prognostic signatures that predict increased risk of recurrence or death is important to guide the selection of appropriate therapies for stage II CRC.

Research has indicated that the tumor immune microenvironment is inextricably linked to tumorigenesis and development, as in stage II CRC, and that immune-related gene (IRG) signatures may indicate immune dysregulation in the immune microenvironment of stage II CRC (Fridman et al., 2012; Gessani and Belardelli, 2019; Tian et al., 2020; Wang J. et al., 2020). Therefore, the molecular signature of IRGs may be valuable as a prognostic biomarker of stage II CRC. Prognostic signatures are commonly used in clinical practice, and gene signature based on large-scale gene expression datasets has been extensively studied in various cancers (Kulasingam and Diamandis, 2008). The construction of prognostic gene signature may help effectively stratify patients and develop personalized treatment strategies (Li et al., 2020). Indeed, various prognostic IRG signatures have been reported in multiple cancer types. For example, a seven IRGs signature for predicting survival in patients with hepatocellular carcinoma was constructed based on the Cancer Genome Atlas (TCGA) (Hu et al., 2020). Similar prognostic signatures based on IRGs have been reported for cervical cancer (Yang S. et al., 2019), ovarian cancer (Shen et al., 2019), papillary thyroid cancer (Lin et al., 2019), invasive ductal cancer (Bao et al., 2019), lung cancer (Song et al., 2019), and gastric cancer (Yang W. et al., 2019). Although these studies highlight the efficacy of prognostic IRG signatures in predicting survival, reliable prognostic signatures based on IRGs have rarely been used to predict the prognosis of patients with stage II CRC.

In the present study, we aimed to develop an IRG signature (IRGCRCII), for predicting prognosis in patients with stage II CRC. After construction of the signature, internal and external cohorts were combined to verify its accuracy and effectiveness. We then built a nomogram based on the IRGCRCII and clinicopathological characteristics, with the aim of clinical practicality. Subsequently, we investigated the relationship between the IRGCRCII signature and the clinicopathological characteristics. Based on the signature, we further performed gene set enrichment analysis (GSEA), tumor mutational burden (TMB) analysis, and tumor-related transcription factor (TF) regulatory network analysis. In addition, we analyzed the correlation between the signature and immune cell infiltration. Importantly, the expression of genes in IRGCRCII was also verified utilizing tissues from 30 patients with stage II CRC and multiple databases to ensure the accuracy and replicability of the bioinformatics results. This IRGCRCII signature may reflect the dysregulation of the immune microenvironment and aid in the prediction of disease-free survival (DFS) in patients with stage II CRC.

## Materials and Methods

### Data Acquisition

Gene expression profiles (data level 3) and related clinical data for patients with CRC were collected from the TCGA data repository^1^ (Ellis et al., 2013). The clinical data included age, sex, tumor stage, T stage, chemotherapy, survival period, and survival status. Patients with stage II CRC were identified in accordance with the 8th edition of the American Joint Committee on Cancer. In addition, stage II CRC samples in the GSE39582 microarray dataset were downloaded from the Gene Expression Omnibus (GEO) database as an external validation cohort^2^ (Marisa et al., 2013). A list of immune-related genes (IRGs) was obtained from the ImmPort database^3^, the largest accessible human immunology database. It offers raw data and protocol exchanges between basic, clinical, and translational research (Bhattacharya et al., 2014).

### Transcriptome Data Processing and Differential Analysis

Transcriptome data were processed using the R package “limma” (Ritchie et al., 2015), filtering out genes with too low or no expression in majority of samples. Eligible genes were then subjected to differential expression analysis between tumor samples and normal samples with the filtering criteria of false discovery rate < 0.01 and | log_2_ fold change (FC)| > 1 (Ping et al., 2020; Wang J. et al., 2020). The obtained differential genes were then intersected with the IRGs downloaded from the Immport database to obtain differentially expressed IRGs.

### Gene Ontology (GO) and Kyoto Encyclopedia of Genes and Genomes (KEGG) Analyses for Differentially Expressed IRGs

In order to gain further insight into the roles of differentially expressed IRGs in biological functions, cellular localization, and different biological pathways, GO and KEGG enrichment analyses of differentially expressed IRGs were performed using the R package “clusterprofiler” (Yu et al., 2012). Results were visualized using the R packages “goplot” and “enrichplot” (Walter et al., 2015), and statistical significance was set at *p* < 0.05.

### Identification and Validation of the Prognostic Signature

Data of patients with complete clinical information were included in the prognostic analysis. A mechanistic learning approach was used to divide the 201 patients with stage II CRC from the TCGA dataset into a training cohort (*n* = 141) and a test cohort (*n* = 60) at a ratio of 7:3. This process was implemented using the R package “caret.” The development of the prognostic IRG signature was based on the data of the training cohort. The test cohort and the total cohort from the TCGA dataset were used as internal validation cohorts, while GSE39582 (*n* = 265) was used as an external validation cohort to evaluate the effectiveness of the prognostic signature.

Univariate Cox regression analysis was used to determine survival IRGs with a threshold value of *p* < 0.05. Next, the least absolute shrinkage and selection operator (LASSO) Cox penalized regression model was performed in order to minimize overfitting, further narrow the range of IRGs from univariate Cox regression analysis, and identify the IRGs most relevant to survival, using the R package “glmnet” (Friedman et al., 2010). Multivariate Cox regression analysis was performed to construct a prognostic IRG signature in stage II CRC, namely IRGCRCII. Stepwise regression was used to introduce Akaike information criterion (AIC) into the multivariate analysis, in which one variable at a time was removed successively to keep reducing the AIC until the smallest AIC value was selected, thereby obtaining the optimal model (Vrieze, 2012). The IRGCRCII risk score was calculated for each patient according to the coefficient and expression of each gene in the signature, as follows: IRGCRCII risk score = $\sum_{i=1}^{k}\beta \,i\,S\,i$ (*k*: the number of genes incorporated into the signature; β*i*: the coefficient for each gene; *Si*: the gene expression level) (Zhang et al., 2020). Using the median IRGCRCII risk score as the cutoff value, the patients in the training cohort were divided into high-risk and low-risk groups. Patient survival was analyzed using Kaplan-Meier and log-rank tests. The specificity and sensitivity of the risk score in predicting 1-, 3-, and 5-years DFS were evaluated based on the area under the curve (AUC) of the receiver operating characteristic (ROC) analysis using the R package “survival ROC.” Furthermore, the IRGCRCII signature was further validated using the test cohort and total cohort, as well as the external validation cohort (GSE39582).

### Association Between the IRGCRCII Signature and Clinicopathological Characteristics

The correlation between patient survival and clinicopathological characteristics, including age, sex, T stage, chemotherapy, and risk scores, was determined utilizing univariate Cox regression analysis. Multivariate Cox regression analysis was used to determine the independent prognostic factors of patients with stage II CRC. At the same time, a nomogram was constructed using the Cox regression coefficients with the R package “rms,” and its calibration curves were drawn with R package “regplot.”

### GSEA and TMB Analysis

In order to reveal the biological characteristics based on IRGCRCII, GSEA (version 4.1.0) software was used to analyze the enrichment of genes in the high-risk and low-risk groups in KEGG pathways (Subramanian et al., 2005). The enrichment *p*-values were obtained by simulating 1,000 random gene set arrangements and the threshold for statistical significance was defined as *p* < 0.05. In addition, mutation data were downloaded from the TCGA website and TMB scores were calculated.

### Construction of a TF Regulatory Network

The TF data were downloaded from the Cistrome Cancer database^4^ (Mei et al., 2017). This database combines publicly accessible chromatin profiling data with TCGA data via a systematic modeling method to analyze the transcriptional and epigenetic factors that control aberrant patterns of gene expression in cancer (Mei et al., 2017). TFs meeting the conditions of *p* < 0.05 and | log_2_ FC| > 1 were considered as differentially expressed TFs. Correlation coefficients > 0.4 and *p* < 0.05, were used as thresholds for the correlation analysis between differentially expressed TFs and the immune genes in the IRGCRCII signature (Li et al., 2020). Eventually, the immunoregulation network was displayed using Cytoscape visualization software (Reimand et al., 2019).

### Evaluation of Tumor-Infiltrating Immune Cells

To estimate the abundance of immune cells in stage II CRC samples, gene expression data were processed using the CIBERSORT web portal^5^ (Gentles et al., 2015). The leukocyte gene signature matrix was obtained using the R package “cibersort. R,” which contains 22 leukocyte subtypes. The perm was set to 1,000, which is the number of permutations used when calculating the *p*-value. Samples with *p* < 0.05 were considered qualified and included for correlation analysis between immune cells and the immune genes in the IRGCRCII signature.

### Clinical Specimens

Thirty pairs of primary tumors and matched paired adjacent normal tissues from patients with stage II CRC diagnosed by pathological examination were obtained from the tissue bank of the Sixth Affiliated Hospital of Sun Yat-sen University in Guangzhou, China. These patients did not receive any preoperative chemotherapy, radiotherapy, or immunotherapy. All patients provided informed consent, and the study was approved by the Medical Ethics Committee of the Sixth Affiliated Hospital of Sun Yat-sen University, Guangzhou, China (no. 2021ZSLYEC-006).

### Quantitative Reverse Transcriptase PCR (qRT-PCR) Analysis

Quantitative reverse transcriptase-PCR (qRT-PCR) was used to quantify the expression of immune genes in IRGCRCII signature in clinical specimens. In accordance with the manufacturer’s instructions, total RNA from the above mentioned 60 tissue samples was extracted using TRIzol Reagent (Invitrogen, United States), and the OD260/OD280 of RNA was detected using a NanoDrop 2000 spectrophotometer (Thermo Fisher Scientific, United States). Only when the OD260/OD280 of RNA was between 1.8 and 2.0, was the RNA used for subsequent reverse transcription with a reverse transcription kit (FSQ-301, Toyobo, Japan) (Huang et al., 2020). Reverse transcription was performed in a 10-μL reaction volume using the Applied Biosystems 7500 Real-time PCR system with SYBR Green Real-time PCR Master Mix (QPK-201, Toyobo, Japan). The relative expression of each gene in IRGCRCII was calculated after normalization to glyceraldehyde phosphate dehydrogenase. Primer sequences are listed in Table 1.

**TABLE 1:** The sequences of primers used in real-time PCR.

Gene	Sequence (5′- 3′)
CCL28	FOR: AATGCAGCAGAGAGGACTCG
	REV: GGCAGCTTGCACTTTCATCC
FGF18	FOR: ACCAGCAAGGAGTGTGTGTT
	REV: CGTCGTGTACTTGAAGGGCT
GAPDH	FOR: GGAGCGAGATCCCTCCAAAAT
	REV: GGCTGTTGTCATACTTCTCATGG
IL23A	FOR: GCTTCATGCCTCCCTACTGG
	REV: TGAGTGCCATCCTTGAGCTG
LIF	FOR: CCCAACAGCAAGACGAGGAT
	REV: GAGATGAGGTGATGGGCGAG
SLIT2	FOR: ATTCCGTTGTTCAGGTACAGAAGAT
	REV: GGGAATGTGCTCCGGGATT
VGF	FOR: TGAAGCCGGAGCGAGCTA
	REV: GAGAGGTGGAGAGGAGGGTC

**FOR, forward primer; REV, reverse primer; GAPDH: glyceraldehyde phosphate dehydrogenase.**

### Immunohistochemistry (IHC)

Four-micron tissue sections were cut from the formalin-fixed, paraffin-embedded tissue blocks of 60 tissue samples. After deparaffinization with dimethylbenzene and rehydration with a graded alcohol series, the sections were incubated in a humidified container with antibodies against FGF18, LIF, IL23A, and SLIT2 at 4°C overnight, followed by incubation with appropriate secondary antibodies for 30 min at 25 ± 5°C. The sections were then stained with 3,3-diaminobenzidine tetrahydrochloride with 0.05% H_2_O_2_ for 3 min for visualization. Fixed positive and negative controls were evaluated in each experiment to control for staining variability among batches of experiments. The immunoreactivity-scoring system (HSCORE, scale 0−3) was used for the semi-quantitative assessment of protein levels in tissues (Liu et al., 2018). Briefly, staining intensity was graded as follows: 0, absence; 1, weak; 2, moderate; 3, strong. The HSCORE was calculated using the following formula: HSCORE = ΣPi × i, where i is the staining intensity and Pi is the percentage of corresponding cells at each level of intensity. Each data point reflected the mean score of two experienced pathologists who were blinded to all clinicopathological variables.

### Multidimensional External Validation

To minimize cohort bias, several databases, including Oncomine (Rhodes et al., 2004), Cancer Cell Line Encyclopedia (Ghandi et al., 2019) and the Human Protein Atlas (Uhlén et al., 2015) were used to detect the expression of immune genes in the IRGCRCII signature and their proteins at tissue and cellular levels.

### Statistical Analysis

All statistical analyses were performed using R software (version 4.0.3) and GraphPad Prism (version 8.0.1). The *Wilcoxon test* was used to compare the two independent nonparametric samples. The chi-square test was used to compare categorical variables. Spearman’s correlation analysis was performed to describe the correlation between quantitative variables without normal distributions. Univariate and multivariate Cox regression analyses were performed to identify independent prognostic factors, and forest plots were created using the R package “forestplot” to display *p*-values, HRs, and 95% CIs for each variable. DFS was defined as the time interval from initial surgical resection to recurrence or death, whichever occurred first (Sargent et al., 2009). Statistical significance was set at *p* < 0.05.

## Results

### Identification of Differentially Expressed IRGs

**FIGURE 1:**
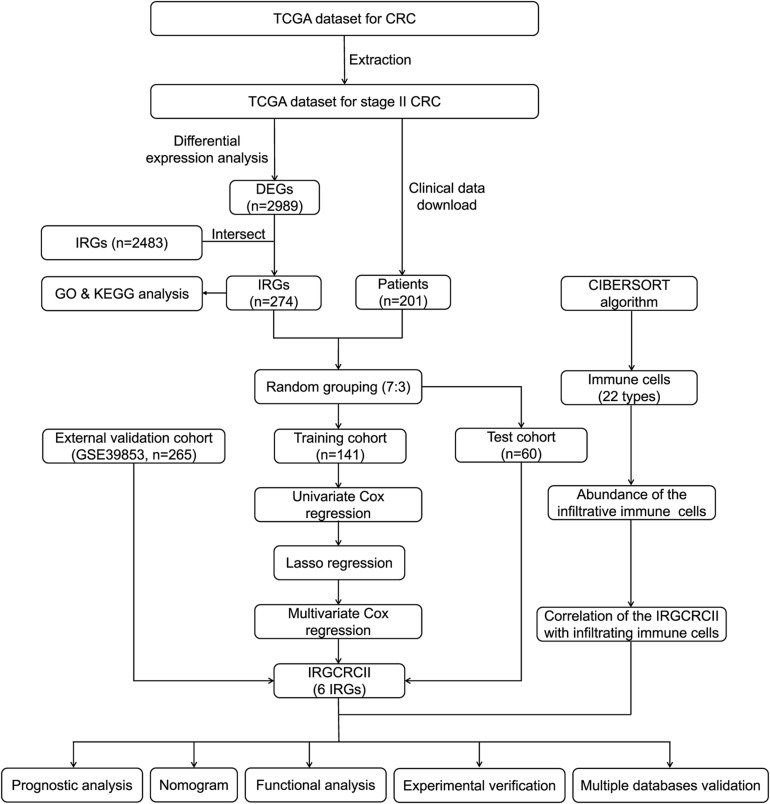
Analysis and design flow chart. CRC, colorectal cancer; DEGs, differentially expressed genes; IRGs, immune-related genes; GO, Gene Ontology; KEGG, Kyoto Encyclopedia of Genes and Genomes; LASSO, the least absolute shrinkage and selection operator method; TCGA, The Cancer Genome Atlas.

**FIGURE 2:**
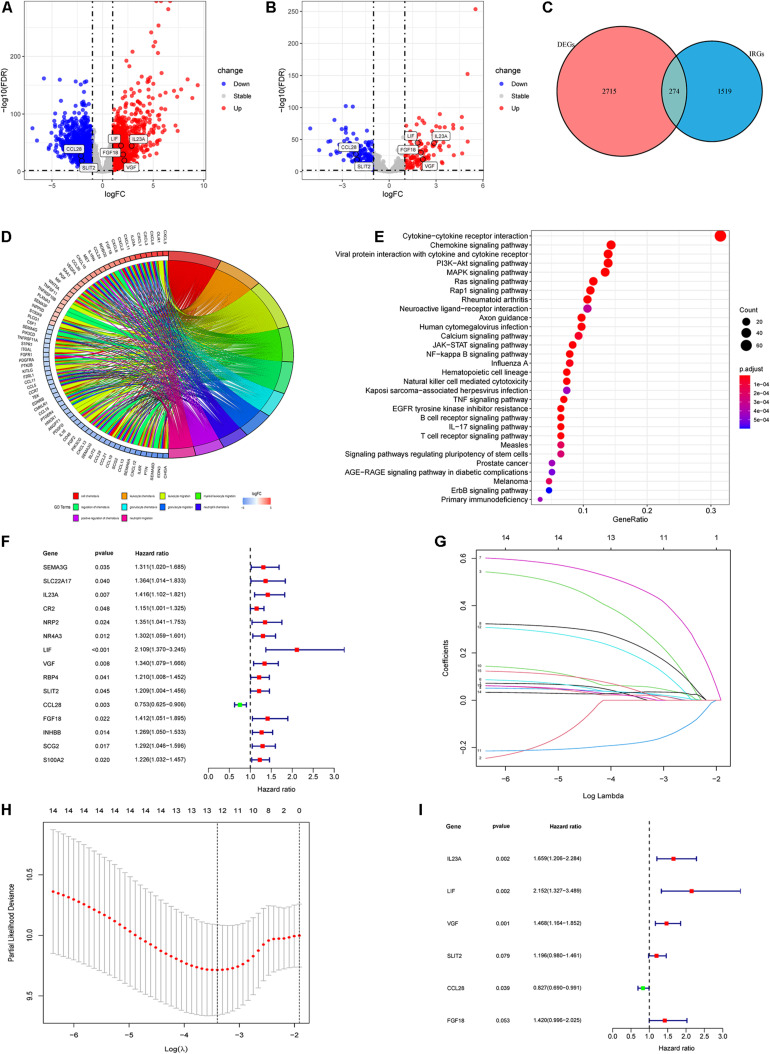
Identification of differentially expressed IRGs and construction of the IRGCRCII model. The volcanic map of DEGs **(A)** and differentially expressed IRGs **(B)** between stage II CRC and normal colorectal tissue, where red represents upregulation and blue represents downregulation, *p* < 0.01, | log_2_ FC| > 2. Venn diagram for the intersections between DEGs and IRGs **(C)**. GO and KEGG pathway enrichment analysis of differentially expressed IRGs **(D,E)**. A forest map showing the relationship between differentially expressed IRGs and DFS in the training cohort **(F)**. Tenfold cross-validation for tuning parameter (lambda) selection in the LASSO model based on minimum criteria for DFS **(G)**. The LASSO coefficient profiles of survival-related IRGs. The dotted line indicates the value chosen by tenfold cross-validation **(H)**. Forest plot of IRGs based on multivariate Cox regression analysis **(I)**. *p* < 0.05. CRC, colorectal cancer; DEGs, differentially expressed genes; DFS, disease-free survival; GO, Gene Ontology; KEGG, Kyoto Encyclopedia of Genes and Genomes; LASSO, the least absolute shrinkage and selection operator method.

After filtering, a total of 466 patients with stage II CRC meeting the criteria were included from the TCGA database (*n* = 201) and the GEO database with the GSE39582 dataset (*n* = 265) (Figure 1 and Table 2). Subsequent differential analysis revealed 2,989 differentially expressed genes (DEGs) that met the conditions of *p* < 0.01 and | log_2_ FC| > 1 (Figure 2A and Supplementary Figure 1A). Then we intersected the 2,989 DEGs and 2483IRGs downloaded from the Immport database yielded 274 differentially expressed IRGs (Figures 2B,C, Supplementary Figure 1B, and Supplementary Table 1). GO analysis revealed that the 274 differentially expressed IRGs were mainly involved in immune and inflammatory responses, such as cell chemotaxis, granulocyte chemotaxis, neutrophil chemotaxis, positive regulation of chemotaxis, and neutrophil migration. KEGG enrichment analyses indicated that the top five significant enrichment pathways were as follows: (1) a cytokine receptor interaction pathway; (2) a chemokine signaling pathway; (3) a pathway involving viral protein interactions with cytokines and cytokine receptors; (4) the PI3K–Akt signaling pathway; and (5) the MAPK signaling pathway (Figures 2D,E).

**TABLE 2:** Patient demographics and clinical characteristics.

Characteristic	TCGA			GEO (GSE39582)
	Total cohort	Training cohort	Test cohort	External Validation cohort
Patients met criteria, n	201	141	60	265
Mean age, yrs	67.19 ± 11.73	67.57 ± 12.42	66.28 ± 9.96	67.70 ± 12.92
**Gender, n**				
Male	111(55.22)	75(53.19)	36(60.00)	157(59.25)
Female	90(44.78)	66(46.81)	24(40.00)	108(40.76)
**T stage, n**				
T3	194(96.52)	131(92.91)	58(96.67)	199(75.09)
T4	13(3.48)	11(7.09)	2(3.33)	51(19.25)
NA	–	–	–	15(5.661)
**Chemotherapy, n**				
Yes	48(23.88)	34(24.11)	14(23.33)	58(21.89)
No	153(76.12)	107(75.89)	46(76.67)	206(77.74)
NA		–	–	1(0.377)
**DFS event, n**				
Yes	50(24.90)	19(13.50)	6(10.00)	95(35.80)
No	151(75.10)	122(86.50)	54(90.00)	170(64.20)

**Data are expressed as n (%) or mean ± standard deviation. yrs, years.**

### Development of the IRGCRCII Signature

**FIGURE 3:**
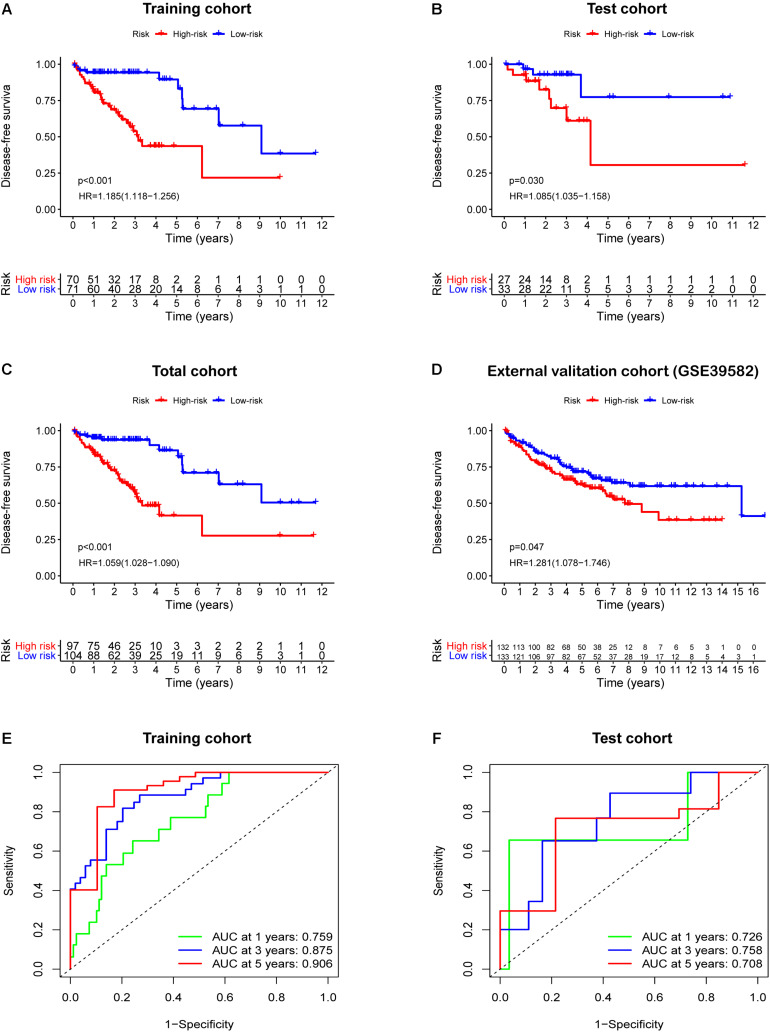
Survival analysis based the IRGCRCII risk score in four cohorts. Kaplan-Meier curves of DFS for patients in the high and low-risk subgroups of the training cohort **(A)**, test cohort **(B)**, total cohort **(C)**, and external validation cohort **(D)**. Time-dependent ROC curves analysis at 1, 3, 5-years DFS of the train cohort **(E)** and the test cohort **(F)**. *p* < 0.05. DFS, disease-free survival; HR, hazard ratio; ROC, receiver operating characteristic.

**FIGURE 4:**
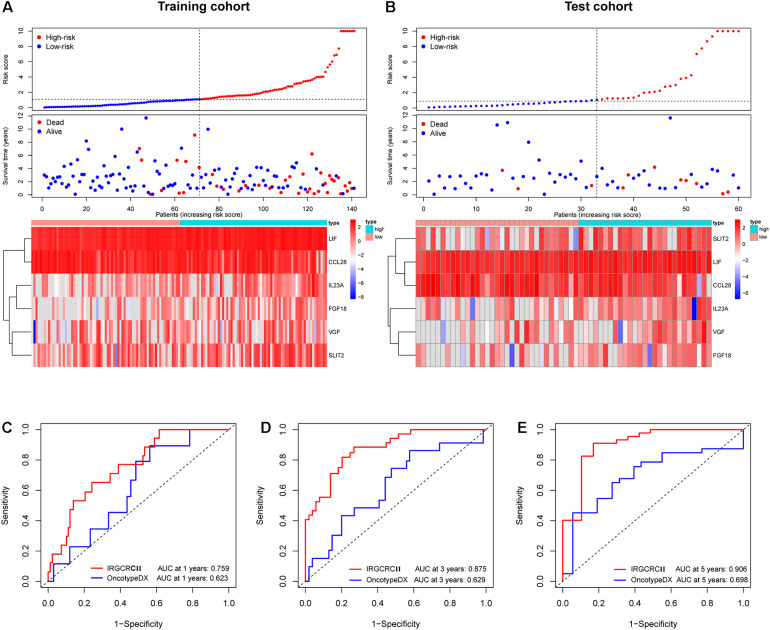
Risk plot for the training and test cohorts and comparison of the IRGCRC and OncotypeDX colon models. Distribution of the risk score, survival status, and gene expression data in the training and test cohorts **(A,B)**. Time-dependent ROC curve analysis at 1- **(C)**, 3- **(D)**, and 5-years. **(E)** DFS for the IRGCRCII model and OncotypeDX colon model in the training cohort. ROC, receiver operating characteristic.

Based on the prominent role played by IRGs in the tumor microenvironment, we explored the prognostic value of differentially expressed IRGs in stage II CRC. Univariate Cox regression analysis of the training cohort yielded 15 IRGs that influenced prognosis (Figure 2F). LASSO Cox regression was then performed to remove two overfitted IRGs (Figures 2G,H). The immune prognostic signature was constructed using a multivariate stepwise regression method. When the minimum AIC score was 290.81, the signature was optimal, involving a total of six IRGs (*CCL28*, *FGF18*, *IL23A*, *LIF*, *SLIT2*, and *VGF*) (Figure 2I and Supplementary Table 2). Then, the coefficient values and expressions of the six IRGs were extracted to calculate the IRGCRCII risk score for each patient using the following formula: IRGCRCII risk score = (−0.190 × level of *CCL28*) + (0.351 × level of *FGF18*) + (0.501 × level of *IL23A*) + (0.766 × level of *LIF*) + (0.179 × level of *SLIT2*) + (0.384 × level of *VGF*). The risk scores were calculated for each patient in the training cohort (*n* = 141) according to the above formula, and the patients were divided into high-risk (*n* = 70) and low-risk (*n* = 71) groups, according to the median risk score of 1.087. The Kaplan-Meier survival curve showed that the high-risk group had worse DFS than the low-risk group (hazard ratio = 1.185, 95% confidence interval = 1.118–1.256, *p* = 0.021, *p* < 0.001) (Figure 3A). ROC analysis of the training cohort showed AUCs of 0.759, 0.875, and 0.906 at 1-, 3-, and 5-years DFS, respectively (Figure 3E). In addition, the risk score curves and survival status plots showed that patients in the high-risk group had a worse prognosis, with more deaths and shorter long-term survival (Figure 4A).

### Validation of the IRGCRCII Signature

The test cohort, total cohort, and external validation cohorts were also divided into high-risk and low-risk groups according to the risk score formula described above. In all three cohorts, Kaplan-Meier survival curves showed that the DFS of the high-risk group was statistically shorter than that of low-risk group (*p* = 0.030, *p* < 0.001, and *p* = 0.047) (Figures 3B–D). ROC analysis of the test cohort revealed AUCs of 0.726, 0.758, and 0.708 at 1-, 3-, and 5-years DFS, respectively (Figure 3F). ROC analysis of the total cohort showed AUCs of 0.755, 0.840, and 0.823 at 1-, 3-, and 5-years DFS, respectively (Supplementary Figure 2A). Furthermore, the risk score curves and survival status plots in the test cohort and total cohort presented similar results to those of the training group (Figure 4B and Supplementary Figure 2B).

### Comparison of the IRGCRCII and OncotypeDX Colon Signatures

To further evaluate the accuracy of the IRGCRCII signature for predicting survival, we compared it with the OncotypeDX colon signature, which is the most widely used gene signature in stage II CRC. Two signatures were used to perform ROC analysis in the training cohort to evaluate the sensitivity and specificity of survival prediction. The AUCs of our IRGCRCII signature were significantly greater than those of the OncotypeDX colon signature at 1 (0.759 vs. 0.623), 3 (0.875 vs. 0.629), and 5 (0.906 vs. 0.698) years, respectively, which indicated that our IRGCRCII signature had better prognostic accuracy (Figures 4C–E).

### IRGCRCII Risk Score as an Independent Prognostic Factor for Stage II CRC

**FIGURE 5:**
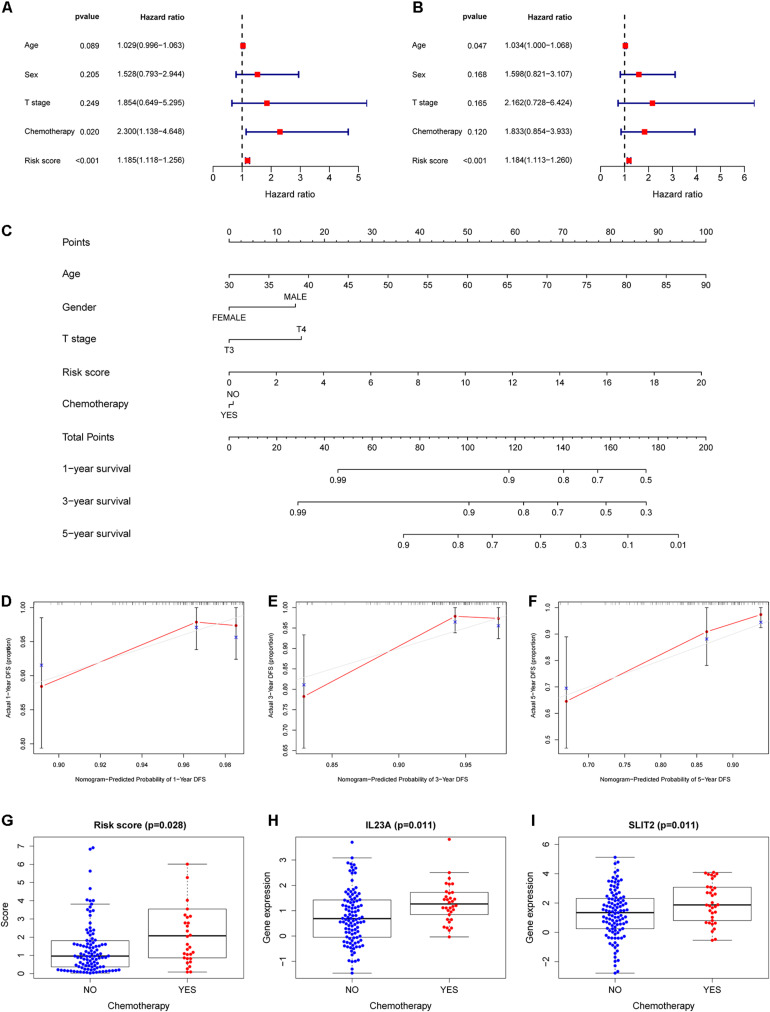
Construction of a nomogram for survival assessment and association between the IRGCRCII model and clinicopathological characteristics. Univariate **(A)** and multivariate **(B)** Cox regression analyses for DFS of stage II CRC in the training cohort. Nomogram constructed by combining clinical characteristics and the IRGCRCII risk score **(C)**. The calibration plots for predicting 1- **(D)**, 3- **(E)**, and 5-years **(F)** DFS. The comparison of risk score **(G)** and expression levels of IL23A **(H)** and SLIT2 **(I)** between chemotherapy group and non-chemotherapy group. *p* < 0.05. CRC, colorectal cancer; DFS, disease-free survival.

To further evaluate the role of the IRGCRCII signature in predicting prognosis, we included the IRGCRCII risk score and some common clinicopathological features such as age, sex, T stage, and chemotherapy in the prognosis-related analysis. In the training cohort, univariate Cox regression analysis showed that chemotherapy, and risk score were significantly associated with patient survival (Figure 5A). Multivariate Cox regression analyses showed that age (hazard ratio = 1.034, 95% confidence interval = 1.000–1.068, *p* = 0.047) and risk score (hazard ratio = 1.184, 95% confidence interval = 1.113–1.260, *p* < 0.001) were independent prognostic factors for stage II CRC (Figure 5B). In the total cohort, we also found that the risk score was an independent prognostic factor after performing univariate and multivariate Cox analyses (Supplementary Figures 3A,B).

### Construction of the Nomogram and Relationships Between the IRGCRCII Signature and Clinicopathological Features

To develop a quantitative method for predicting the prognosis of patients with stage II CRC in clinical settings, we established a nomogram in the training cohort, integrating clinicopathological features and IRGCRCII risk score (Figure 5C). Among them, age had the greatest impact on prognosis, followed by risk score, T-stage, sex, and chemotherapy. The calibration curves for 1-, 3-, and 5-years DFS were close to the standard curve, and the concordance index (C-index) was 0.779, indicating good model performance (Figures 5D–F). We also analyzed correlations between the IRGCRCII risk score and clinical features. As shown in Figure 5G, patients in the chemotherapy group had higher risk scores than those in the non-chemotherapy group. The immune genes *SLIT2* and *IL23A* were also significantly more abundant in the chemotherapy group than in the non-chemotherapy group (Figures 5H,I).

### GSEA, TMB, and TF Regulatory Network Analyses of the IRGCRCII Signature

GSEA was used to evaluate the potential association between the IRGCRCII signature and biological functions in the training cohort. The results showed that 11 KEGG pathways were significantly enriched (*p* < 0.05). The high-risk group exhibited significant enrichment in axon guidance (*p* < 0.001), the GNRH signaling pathway (*p* < 0.001), the MAPK signaling pathway (*p* < 0.001), melanogenesis (*p* < 0.001), vascular smooth muscle contraction (*p* = 0.01), and the VEGF signaling pathway (*p* = 0.03). The low-risk group exhibited significant enrichment in cell cycle functions (*p* = 0.04), DNA replication (*p* = 0.020), homologous recombination (p = 0.01), mismatch repair (*p* = 0.01), and nucleotide excision repair (*p* = 0.02) (Supplementary Figure 4A). Because TMB is closely related to the immunotherapy of colorectal cancer, we calculated TMB scores for each sample with mutations in the training cohort to compare the differences between the high-risk and low-risk groups. However, the results showed that the TMB scores of the high-risk group were not significantly different from those of the low-risk group (Supplementary Figure 4B), indicating that there may be no difference in immunotherapy between the two groups.

In addition, we performed differential expression analysis of 318 TFs, resulting in 66 differentially expressed TFs (*p* < 0.05 and | log_2_ FC| > 1) (Supplementary Table 3). The regulatory relationships between the nine differentially expressed TFs and three genes in the IRGCRCII signature were shown in the network (correlation coefficients > 0.4 and *p* < 0.05) to explore the transcriptional and epigenetic factors controlling aberrant patterns of gene expression in stage II CRC (Supplementary Figure 4C).

### Correlation Between the IRGCRCII Signature and Immune Cell Infiltration

**FIGURE 6:**
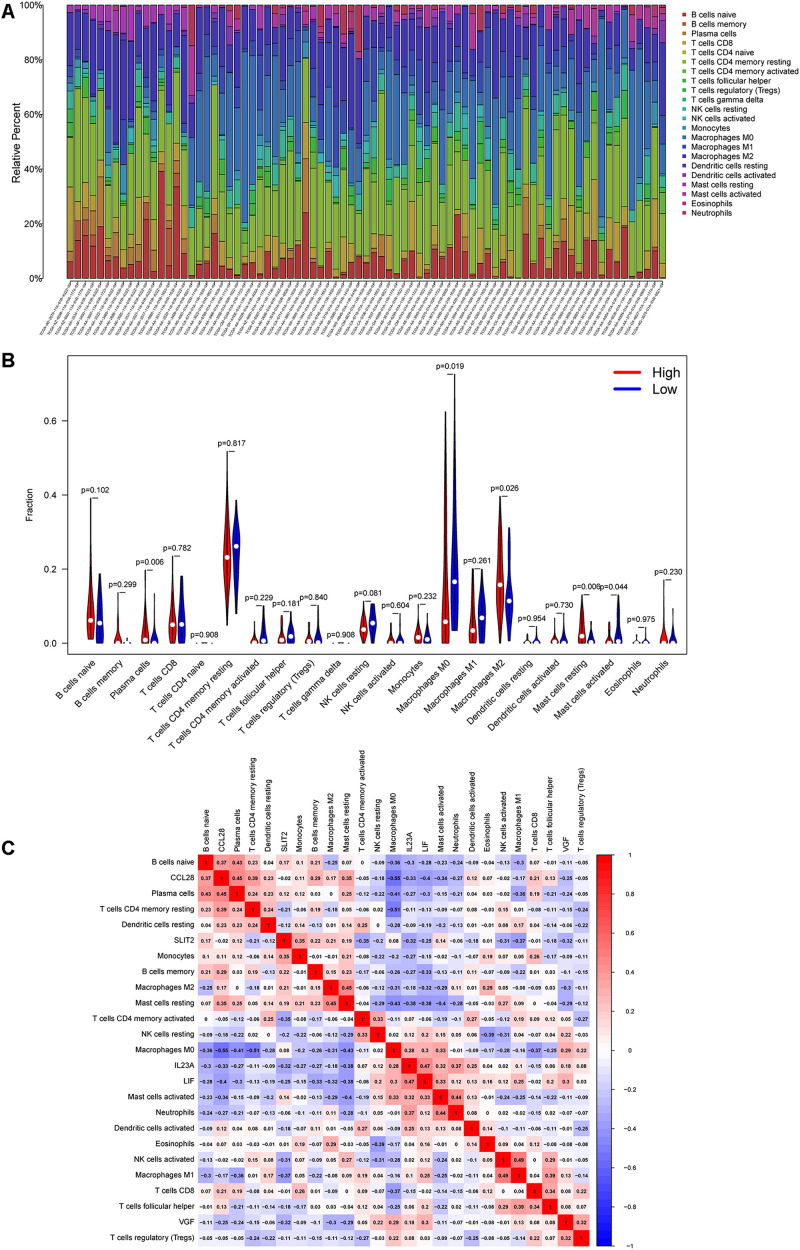
Correlation between the six immune genes in IRGCRCII and immune cell infiltration. The percentage stacked bar chart shows the distribution of the 22 immune cells in the stage II CRC samples from TCGA **(A)**. The violin plots present differences in the abundance of immune cells between the high-risk and low-risk groups. Blue represents the low-risk group, while red represents the high-risk group **(B)**. Correlation matrix of the six immune genes with 22 tumor-infiltrating immune cells. Red represents a positive correlation, while blue represents a negative correlation **(C)**. *p* < 0.05. CRC, colorectal cancer; TCGA, The Cancer Genome Atlas.

Based on a cutoff value of *p* < 0.05, we screened 244 samples from the total cohort and calculated the percentage of the 22 immune cells in each sample. As shown in Figure 6A, the composition of the 22 immune cells varied among the different samples. Violin plots were also used to analyze the differential of immune cells in the high-risk and low-risk groups. The violin plot revealed a significant increase in the proportion of M2 macrophages (*p* = 0.026), plasma cells (*p* = 0.006), and resting mast cells (*p* = 0.006) in the high-risk group when compared to that in the low-risk group. However, M0 macrophages (*p* = 0.019) and activated mast cells (*p* = 0.044) were significantly more abundant in the low-risk group than in the high-risk group (Figure 6B).

As shown in Figure 6C, the co-expression patterns were observed in the correlation analysis between the six immunegenes in the IRGCRCII signature and the tumor-infiltrating immune cells. Using *p* < 0.05 and | correlation coefficients| > 0.3 as thresholds, the analysis revealed that *CCL28* was positively correlated with resting memory CD4+ T cells, while it was negatively correlated with M0 macrophages (*p* = 0.01) (Supplementary Figures 5A,B). *FGF18* was positively correlated with M0 macrophages and negatively correlated with resting memory CD4+ T cells and neutrophils (Supplementary Figures 5C–E). *SLIT2* was positively correlated with memory B cells, M0 macrophages, and monocytes (Supplementary Figures 5F–H). *VGF* was positively correlated with regulatory T cells (Tregs) (*p* = 0.01) and resting NK cells (*p* < 0.01) (Supplementary Figures 5I,J). In addition, the IRGCRCII risk score was negatively correlated with resting memory CD4+ T cells (Supplementary Figure 5K).

### Preliminary Experimental Validation

**FIGURE 7:**
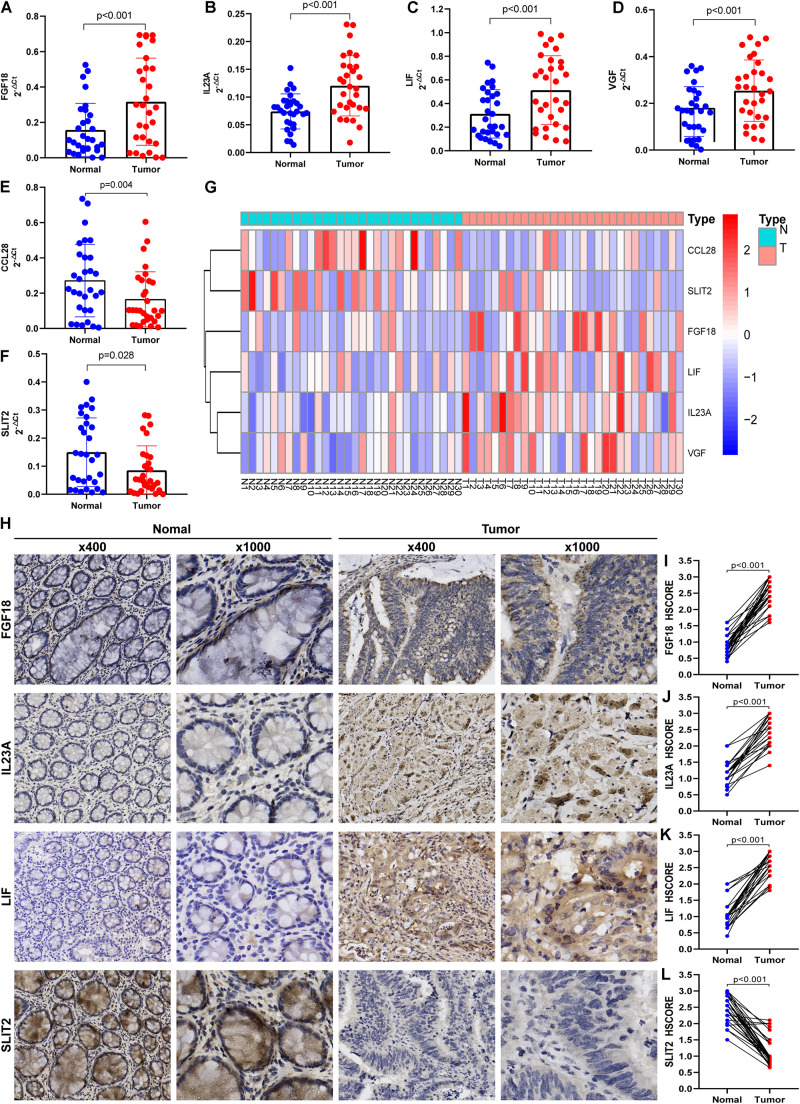
Preliminary clinical specimen validation in 30 pairs of primary Stage II CRC and matched adjacent normal tissues. The gene expressions of *FGF18* **(A)**, *IL23A* **(B)**, *LIF* **(C)**, *VGF* **(D)**, *CCL28* **(E)**, and *SLIT2* **(F)** in tumor and normal tissues were examined via qRT-PCR. The expression levels of the six immune genes in the IRGCRCII are illustrated in a heatmap **(G)**. The IHC assay **(H)** was used to examined the protein expressions of FGF18 **(I)**, IL23A **(J)**, LIF **(K),** and SLIT2 **(L)**. *p* < 0.05. CRC, colorectal cancer; qRT-PCR, quantitative real-time polymerase chain reaction; IHC, immunohistochemistry.

To verify the accuracy of bioinformatics analysis, we examined the expression levels of IRGs in the IRGCRCII signature in 30 pairs of primary tumors and matched adjacent normal tissues. The demographics and clinical characteristics of the 30 patients with stage II CRC are shown in Supplementary Table 4. The results of qRT-PCR were consistent with the bioinformatics analysis described above. Compared using *paired Wilcoxon test*, *FGF18*, *IL23A*, *LIF*, and *VGF* were significantly elevated (*p* < 0.001) (Figures 7A–D), and CCL28 and SLIT2 were significantly downregulated (*p* < 0.05) (Figures 7E,F). The expression levels of the six genes are also illustrated in the heatmap (Figure 7G). The protein expression levels of FGF18, IL23A, LIF, and SLIT2 were examined via immunohistochemistry (IHC) (Figure 7H). The results indicate that the mean HSCORES of FGF18, IL23A, and LIF in tumor tissues were significantly higher than those in normal tissues (*p* < 0.05) (Figures 7I–K), while the opposite trend was observed for SLIT2 (Figure 7L).

### Multidimensional Validation Based on Multiple Databases

To further minimize bias, multiple databases were used to determine the expression of the six immunegenes in the IRGCRCII signature and their protein expression levels at the tissue and cell levels (Table 3). The results from the Oncomine database were completely consistent with the differential analysis above, which showed that *FGF18*, *IL23A*, *LIF*, and *VGF* were highly expressed, while *CCL28* and *SLIT2* were lowly expressed in tumors (Supplementary Figures 6A–F). In the Cancer Cell Line Encyclopedia database, *IL23A* and *LIF* were found to be highly expressed in CRC cell lines. However, *CCL28*, *FGF18*, *SLIT2*, and *VGF* were expressed at low levels in CRC cells (Supplementary Figures 7A–F). In addition, at the protein level, *FGF18*, *IL23A*, *LIF*, and *VGF* stained more deeply in tumor tissues than in normal tissues, while *CCL28* and *SLIT2* were only deeply stained in normal intestinal mucosal tissues according to the Human Protein Atlas (Supplementary Figures 8A–F).

**TABLE 3:** Summary of multidimensional external validation results based on multiple databases.

Database	*FGF18*		*IL23A*		*LIF*		*VGF*		*CCL28*		*SLIT2*		Results
	T	N	T	N	T	N	T	N	T	N	T	N	
Oncomine	↑	NA	↑	NA	↑	NA	↑	NA	↓	NA	↓	NA	*LIF*, *IL23A*, *FGF18*, and *VGF* were highly expressed in tumors, while *CCL28* and *SLIT2* were lowly expressed in tumors
CCLE	↓	NA	↑	NA	↑	NA	↓	NA	↓	NA	↓	NA	At the cellular level, *LIF* and *IL23A* were highly expressed in colorectal cancer cell lines, while *FGF18*, *CCL28*, *VGF*, and *SLIT2* were low expressed in colorectal cancer cell lines
HPA	↑	↓	↑	↓	↑	↓	↑	↓	↓	↑	↓	↑	IHC results showed that LIF, IL23A, FGF18, and VGF stained deeply with antibodies in tumor tissues, while CCL28 and SLIT2 stained deeply with antibodies in normal tissues

**FGF18, and VGF stained deeply with antibodies in tumor tissues, while CCL28 and SLIT2 stainupregulated gene; “↓” was defined as a significantly downregulated gene; “NA” was defined as “Not available.” CCLE, Cancer Cell Line Encyclopedia; HPA, Human Protein Atlas; IHC, immunohistochemistry.**

## Discussion

Despite radical surgical treatment, patients with stage II CRC are still at a high risk of recurrence or death (Al-Temaimi et al., 2016; Ke et al., 2020; Wang K. et al., 2020). Thus, reliable prognostic signatures are urgently needed to predict this increased risk in patients with stage II CRC. To address the issue, we constructed a novel immune gene-derived prognostic signature (IRGCRCII) that includes six immune genes (*CCL28*, *FGF18*, *IL23A*, *LIF*, *SLIT2*, and *VGF*).

The IRGCRCII signature successfully stratified patients with stage II CRC in the training cohort into high-risk and low-risk groups. Our analysis revealed that the high-risk group exhibited worse DFS (*p* < 0.001) than the low-risk group. The AUC values for 1-, 3-, and 5-years DFS of this prognostic signature were 0.759, 0.875, and 0.906, respectively, indicating that the prediction accuracy was high. Notably, our research also combined internal and external validation cohorts to verify the applicability and effectiveness of the IRGCRCII signature in predicting survival. In addition, when compared with the representative known OncotypeDX colon signature, our IRGCRCII signature achieved higher accuracy based on the satisfactory AUCs at 1- (0.759 vs. 0.623), 3- (0.875 vs. 0.629), and 5-years (0.906 vs. 0.698) DFS. Univariate and multivariate Cox regression analyses indicated that the IRGCRCII risk score was an independent prognostic risk factor. We also established a nomogram integrating the IRGCRCII risk score and clinicopathological features to allow colorectal surgeons to assess the risk of postoperative recurrence or death more conveniently. The nomogram performance was quite good after evaluation using the calibration curves and C-index (0.779). Above all, these findings demonstrated that the IRGCRCII signature can be valuable to patients with stage II CRC and colorectal surgeons because it can help evaluate the risk of tumor recurrence or death after surgical treatment and guide clinical treatment decisions.

All six immune genes in the IRGCRCII signature have been reported to be involved in the development and progression of tumors (Shimokawa et al., 2003; Lan et al., 2011; Hwang et al., 2017; Shi et al., 2019; Sun et al., 2019; Yao et al., 2019), which may explain why the IRGCRCII signature is associated with patient prognosis. For example, *CCL28* has previously been identified as part of a prognostic signature that can accurately predict survival in patients with CRC (Sun et al., 2019; Wang J. et al., 2020). Shimokawa et al. (2003) reported that *FGF18* is activated in colon cancers as a direct downstream target of the Wnt signaling pathway. Shi et al. (2019) determined that both pharmacological LIF blockade and genetic LIF deletion markedly slowed tumor progression, mainly by modulating cancer cell differentiation and epithelial-mesenchymal transition (EMT). IL-23R is highly positive in CRC cells, and the IL-23/IL-23R pathway is a potential route facilitating the malignant progression of cancers (Lan et al., 2011). Yao et al. (2019) revealed that SLIT2 can induce tumor metastasis partially through activation of the TGF-β/Smad pathway in CRC. Hwang et al. (2017) demonstrated that high expression of VGF promotes EMT and cancer dissemination. In addition, our TF regulatory network analysis further indicated that TFs, including FOSL, MEIS1, MYH11, and TCF7 were significantly correlated with the immune genes in the IRGCRCII signature, which also affected cancer progression and prognosis. Luo et al. (2018) reported that high expression of FOSL in prostate cancer can accelerate tumor metastasis. Another study found that knockdown of MEIS1 enhances the invasiveness of gastric cancer cells (Qu et al., 2020). Alhopuro et al. (2008) also noted that mutations in MYH11 can contribute to intestinal tumorigenesis. It has also been reported that high expression of TCF7 in perihilarcholangiocarcinoma indicates poor prognosis (Liu et al., 2019).

To understand the potential mechanism by which the IRGCRCII signature affected the prognosis of patients with stage II CRC, we used GSEA to analyze differences in KEGG pathways between the high-risk and low-risk groups. This analysis indicated that six pathways were significantly enriched in the high-risk group, including axon guidance, GnRH signaling, MAPK signaling, melanogenesis, vascular smooth muscle contraction, and VEGF signaling pathways. All six of these pathways have been associated with poor prognosis for CRC (Je et al., 2013; Zhu et al., 2013; Hohla et al., 2014; Sun et al., 2018; Lu et al., 2020; Zhao et al., 2020a), which may provide insight into the molecular mechanisms underlying poor prognosis in the high-risk group.

As one of the key components of the tumor microenvironment, tumor-infiltrating immune cells are significantly associated with the prognosis of patients with CRC (Ge et al., 2019; Peng et al., 2019; Tian et al., 2020). In our study, a newly developed computer-based analysis algorithm, CIBERSORT, was introduced to assess the components of immune cells. We calculated the composition of 22 immune cell types in each sample, and further analysis of the qualified samples showed that M2 macrophages, resting mast cells, and plasma cells were significantly more abundant in the high-risk group than in the low-risk group. Zhao et al. (2020b) demonstrated that M2 macrophage polarization can promote liver metastasis in CRC. Another study reported that mast cell infiltration is inversely correlated with prognosis in patients with lung cancer (Imada et al., 2000). Moreover, proliferation of malignant plasma cells in the bone marrow is a characteristic manifestation of multiple myeloma (Łacina et al., 2020). Above all, the tumor-infiltrating immune cell environment indicates the immune status of patients with cancer, which may account for the difference in survival outcomes between the high-risk and low-risk groups.

In this study, we not only demonstrated the validity and applicability of the IRGCRCII signature for predicting prognosis in patients with stage II CRC through multiple internal and external independent cohorts, but also analyzed the relationship of the signature to clinicopathological features, immune cell infiltration, GSEA, and TMB in depth. Notably, we also verified the expression of the six IRGs included in the IRGCRCII signature and their protein expression levels through qRT-PCR and IHC analyses in 60 clinical specimens. However, there were several limitations to our study. First, this was a retrospective analysis performed using public databases, and selection bias is difficult to avoid in such settings. In an attempt to address this, we used multiple internal and external cohorts to verify the accuracy of the signature. Second, although we performed qPCR and immunohistochemistry in clinical specimens, additional *in vitro* and *in vivo* functional experiments need to be performed to further understand the biological role of the IRGCRCII signature in stage II CRC. Therefore, further validations using multicenter prospective data and experiments are required before the signature can be applied in clinical practice.

## Conclusion

In our study, we developed and validated a novel immune prognostic signature based on six immune-related genes in patients with stage II CRC, which not only predicted survival in multiple internal and external cohorts but also reflected immune dysregulation in the tumor microenvironment.

## Data Availability Statement

The datasets presented in this study can be found in online repositories. The names of the repository/repositories and accession number(s) can be found in the article/Supplementary Material.

## Ethics Statement

The studies involving human participants were reviewed and approved by the Medical Ethics Committee of the Sixth Affiliated Hospital of the Sun Yat-sen University, Guangzhou, China. The patients/participants provided their written informed consent to participate in this study. Written informed consent was obtained from the individual(s) for the publication of any potentially identifiable images or data included in this article.

## Author Contributions

XL, MX, and SY analyzed the data, conducted the experiments, designed the study, and wrote the manuscript. ZX and CM conducted the experiments and critically revised the manuscript. FZ, HC, and LJ analyzed the data and critically revised the manuscript. PL and LL participated in the conception of the study, designed the study, and revised the manuscript critically. All authors contributed to the article and approved the submitted version.

## Conflict of Interest

The authors declare that the research was conducted in the absence of any commercial or financial relationships that could be construed as a potential conflict of interest.

## Footnotes

1 https://www.cancer.gov

2 www.ncbi.nlm.nih.gov/geo

3 https://immport.niaid.nih.gov

4 http://cistrome.org/CistromeCancer

5 http://cibersort.stanford.edu/

## Abbreviations

AUC: area under the receiver operating characteristic
AIC: Akaike Information Criterion
CRC: colorectal cancer
DEGs: differentially expressed genes
DFS: disease-free survival
GEO: Gene Expression Omnibus
GO: Gene Ontology
GSEA: gene set enrichment analysis
IRGs: Immune-related genes
KEGG: Kyoto Encyclopedia of Genes and Genomes
LASSO: the least absolute shrinkage and selection operator method
qRT-PCR: Quantitative reverse transcriptase-PCR
ROC: receiver operating characteristic
TF: transcription factor
TCGA: the Cancer Genome Atlas
TMB: tumor mutational burden.
