# Supplementary material for: Identification and Validation of a Six Immune-Related Genes Signature for Predicting Prognosis in Patients With Stage II Colorectal Cancer
Source: Front Genet. 2021 May 4;12:666003. doi: 10.3389/fgene.2021.666003 (PMC8129521; doi:10.3389/fgene.2021.666003)
Supplement: Supplementary file 1 [file Data_Sheet_1.docx]

Supplementary Material

# Supplementary Figures and Tables

## Supplementary Figures


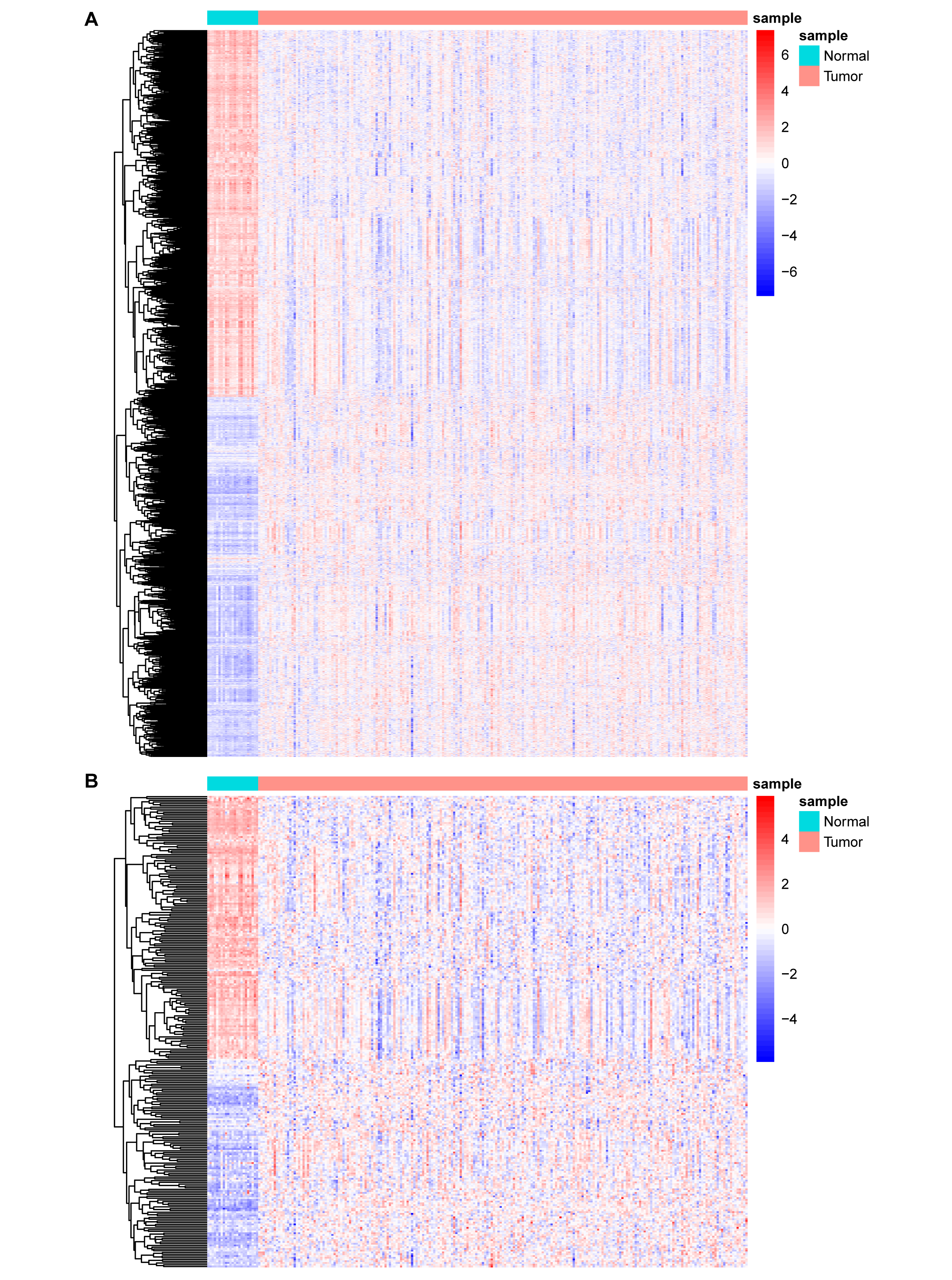
**Supplementary Figure 1**. Heatmap of DEGs between stage II CRC and normal tissues. Heatmap demonstrating the 2,989 DEGs **(A)** and the 274 differentially expressed DEGs **(B)**. *p*<0.01, |log2FC| > 2. Abbreviation: CRC, colorectal cancer; DEG, differentially expressed gene.


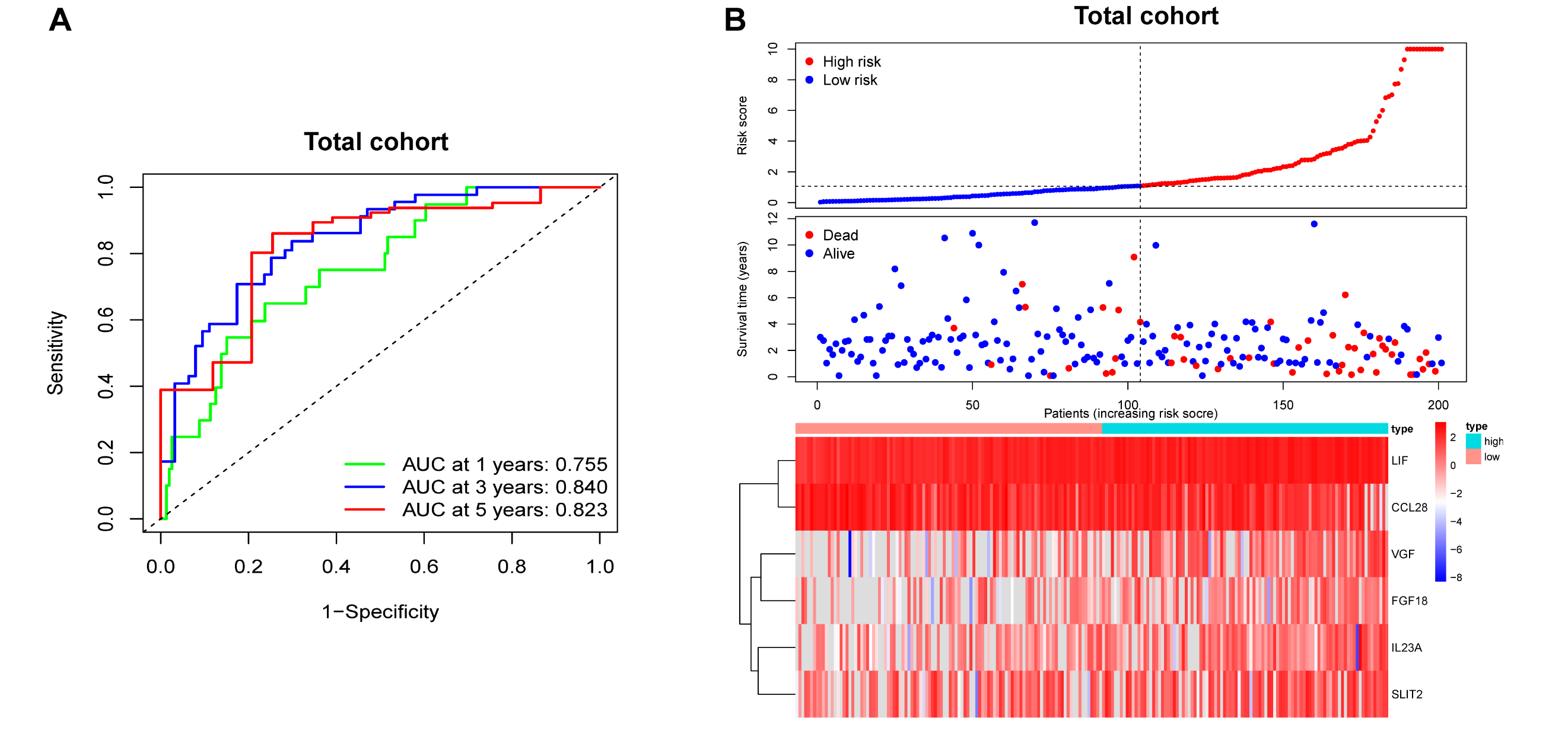
**Supplementary Figure 2**. Validation of the IRGCRCII model in the total cohort. Time-dependent ROC curve analysis at 1-, 3-, and 5-year DFS in the total cohort (AUCs = 0.755, 0.840, 0.823) **(A)**. Distribution of the risk score, survival status, and gene expression data in the total cohort **(B)**. Abbreviation: DFS, disease-free survival; ROC, receiver operating characteristic.


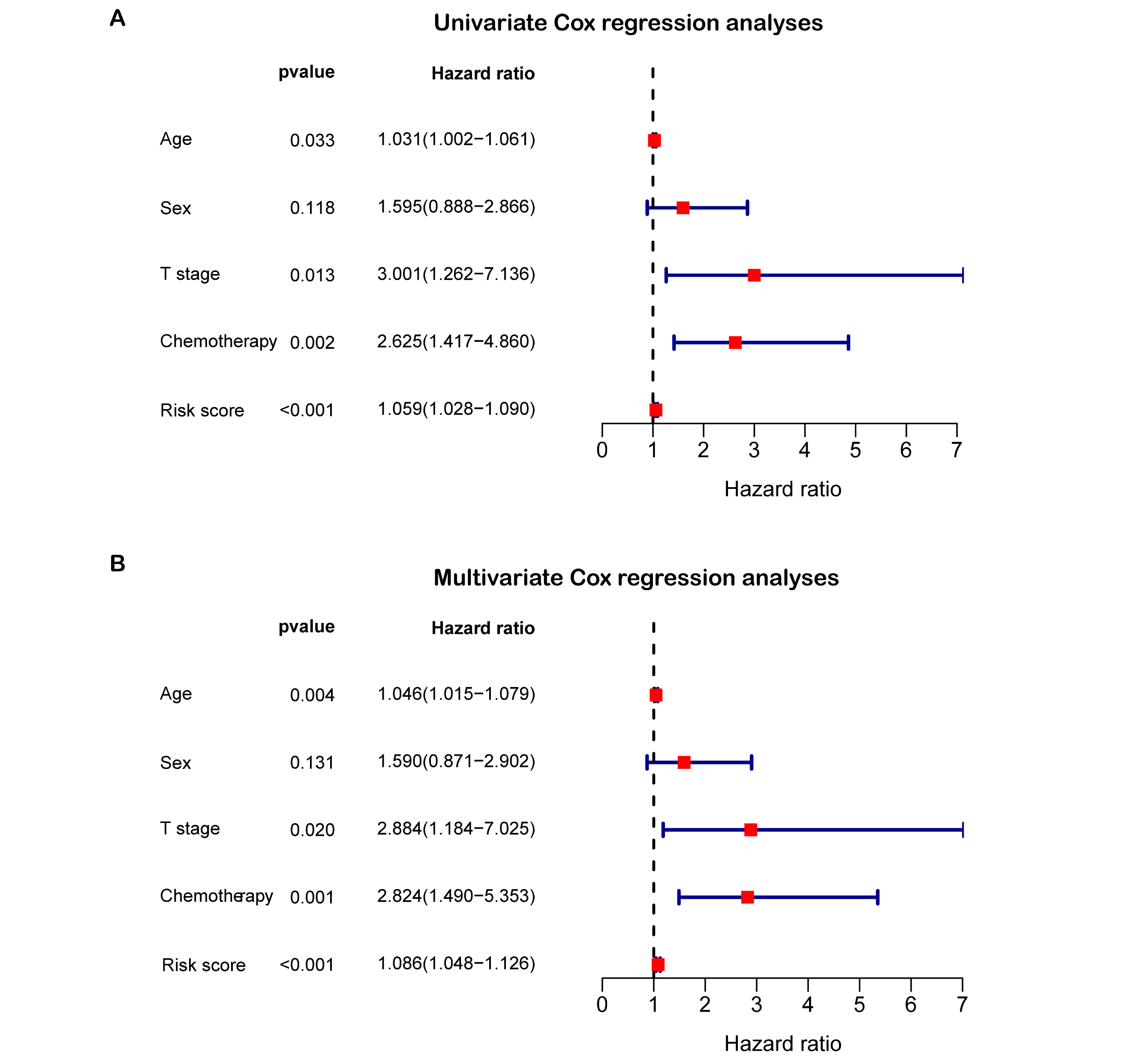


**Supplementary Figure 3**. Univariate **(A)** and multivariable **(B)** Cox regression analyses including the IRGCRII risk score and some common clinicopathological features such as age, sex, T stage, and chemotherapy for the total cohort. *p* < 0.05.


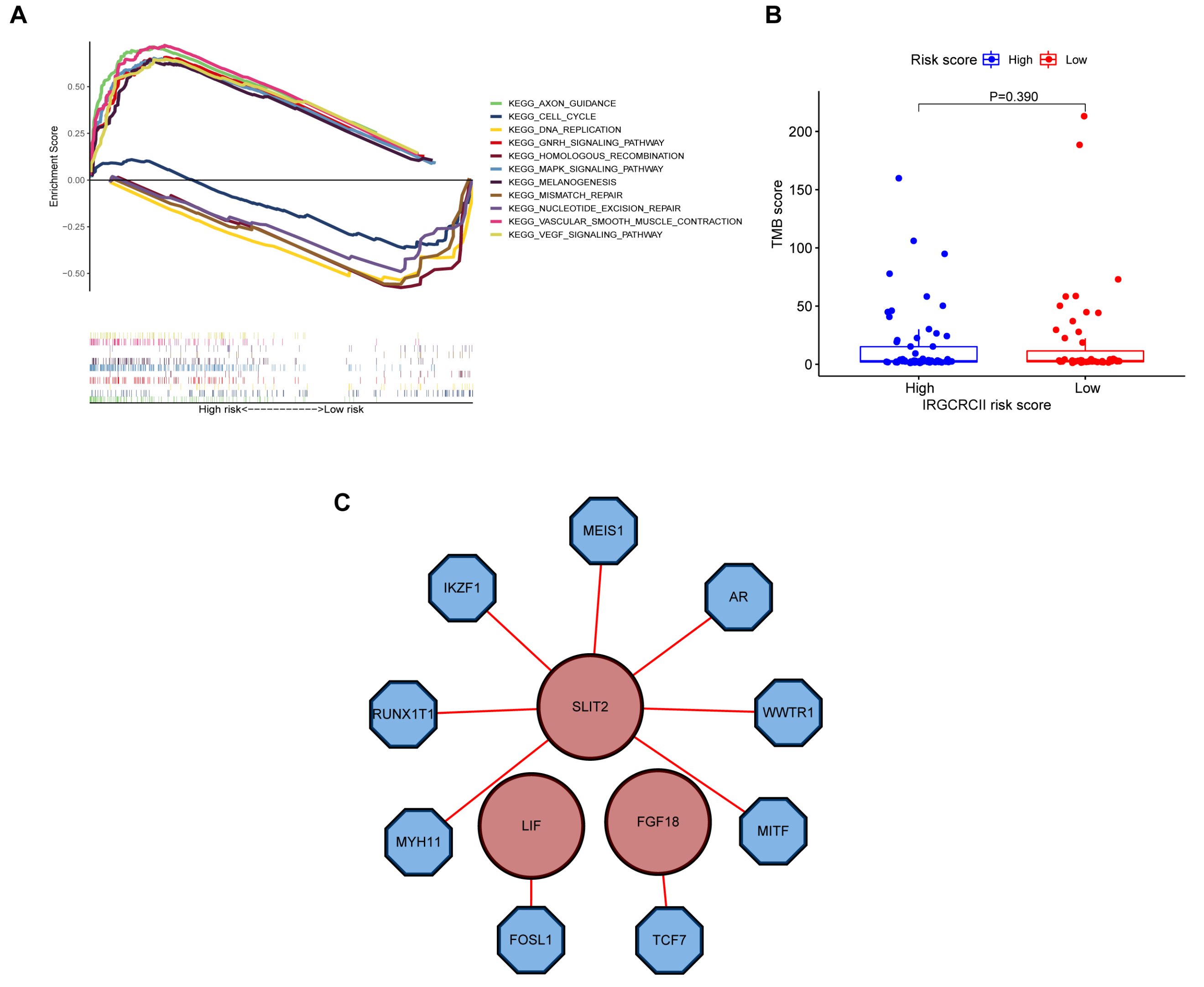


**Supplementary Figure 4**. GSEA was performed on genes from high-risk and low-risk groups with 1,000 permutations per run **(A)**. Comparison of TMB scores in the high-risk and low-risk groups **(B)**. Regulatory networks between nine differentially expressed TFs and 3 IRGs in the IRGCRII model **(C)**. The circle represents the IRGs; the octagon represents the TFs; and the red line represents positive regulation. *p* < 0.05. Abbreviation: IRG, differentially expressed immune-related gene; GSEA, gene set enrichment analysis; TMB, tumor mutational burden, TF, transcription factor.


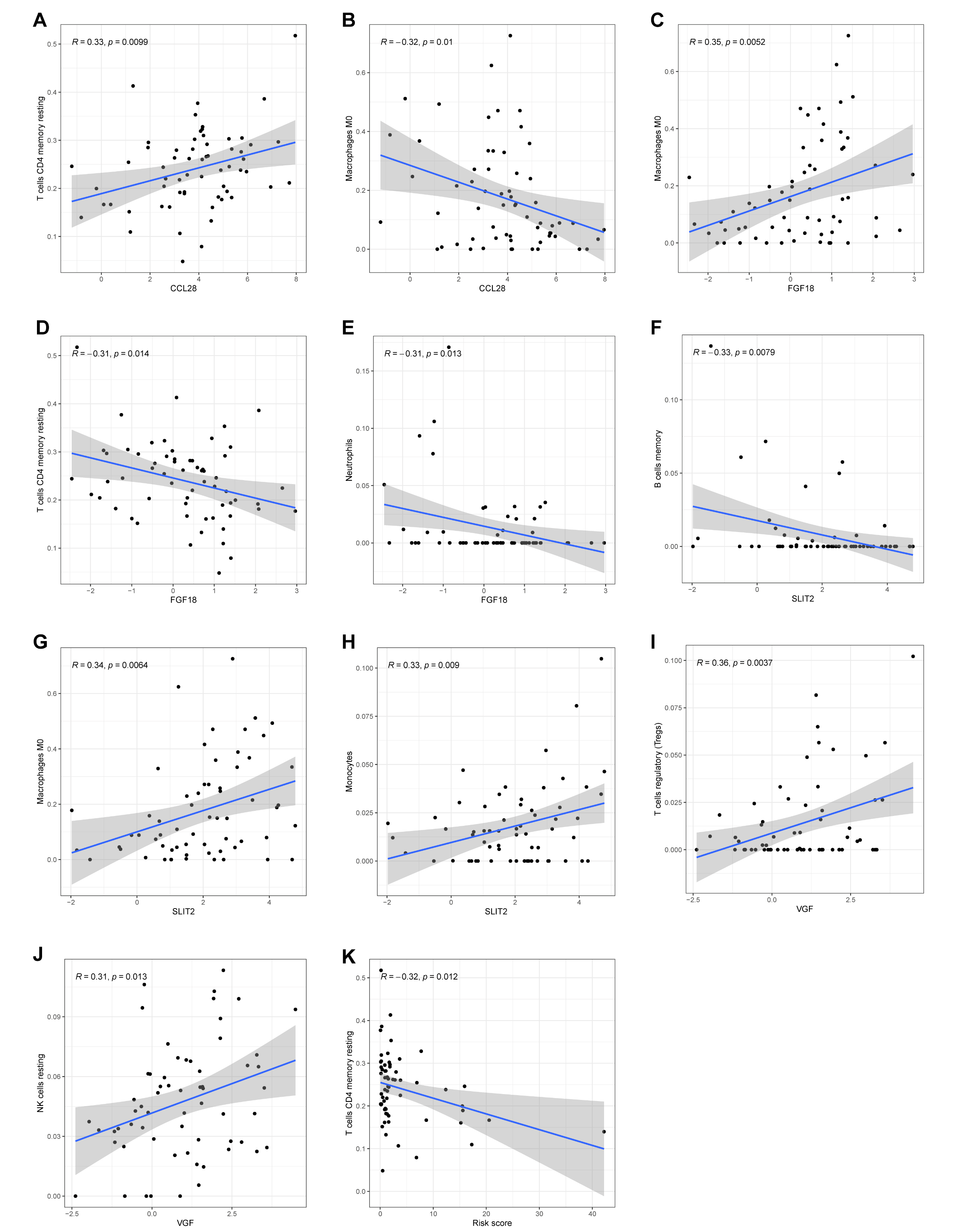


**Supplementary Figure 5**. Correlation analysis between the six IRGs in the IRGCRCII model and tumor-infiltrating immune cells **(A-K)**. *p* < 0.05. Abbreviation: IRGs, immune-related gene.


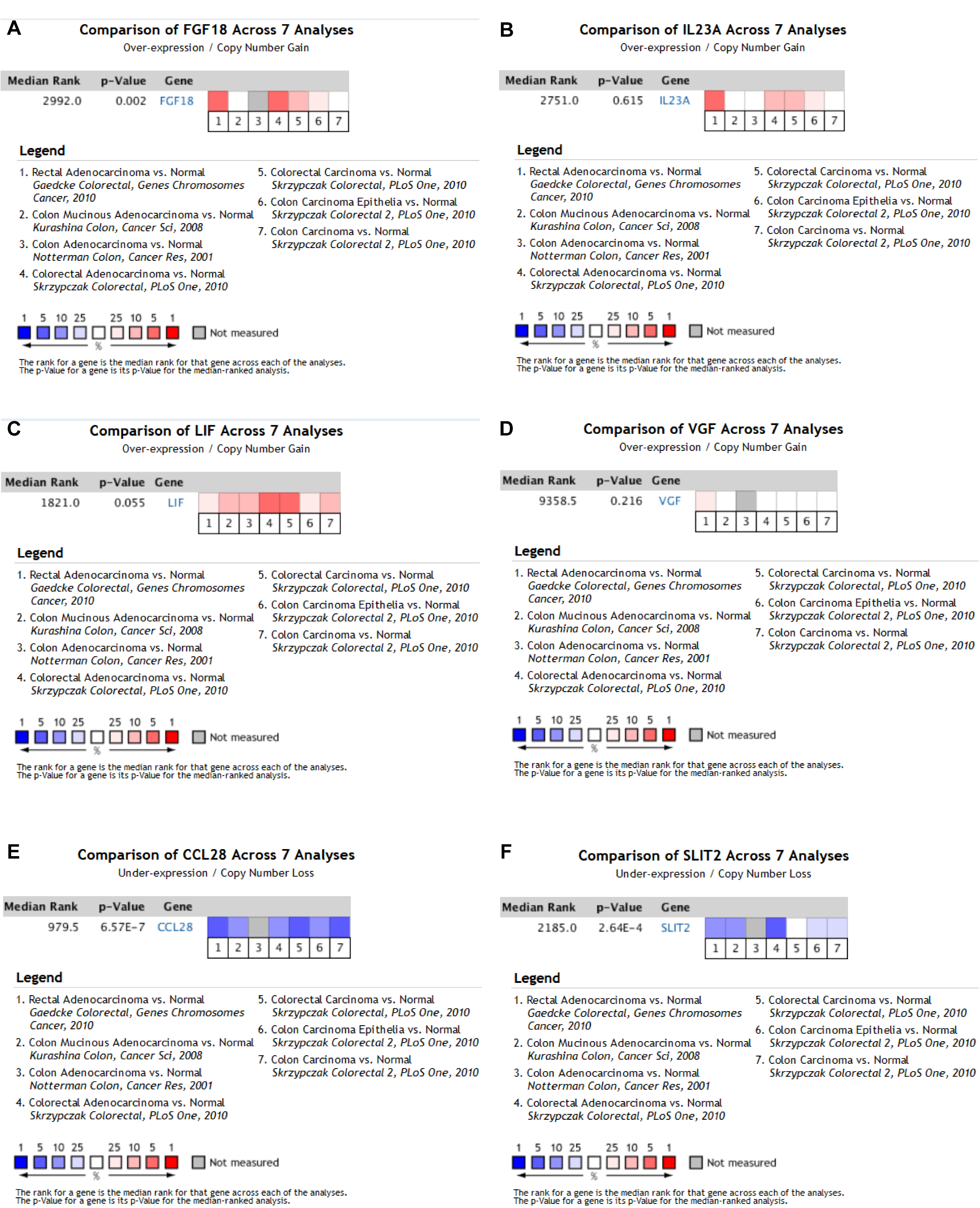


**Supplementary Figure 6**. The expression of *FGF18* **(A)**, *IL23A* **(B)**, *LIF* **(C)**, *VGF* **(D)**, *CCL28* **(E)**, and *SLIT2* **(F)** in multiple CRC tumor studies was validated at the transcriptomic level using the Oncomine database. Abbreviation: CRC, colorectal cancer.


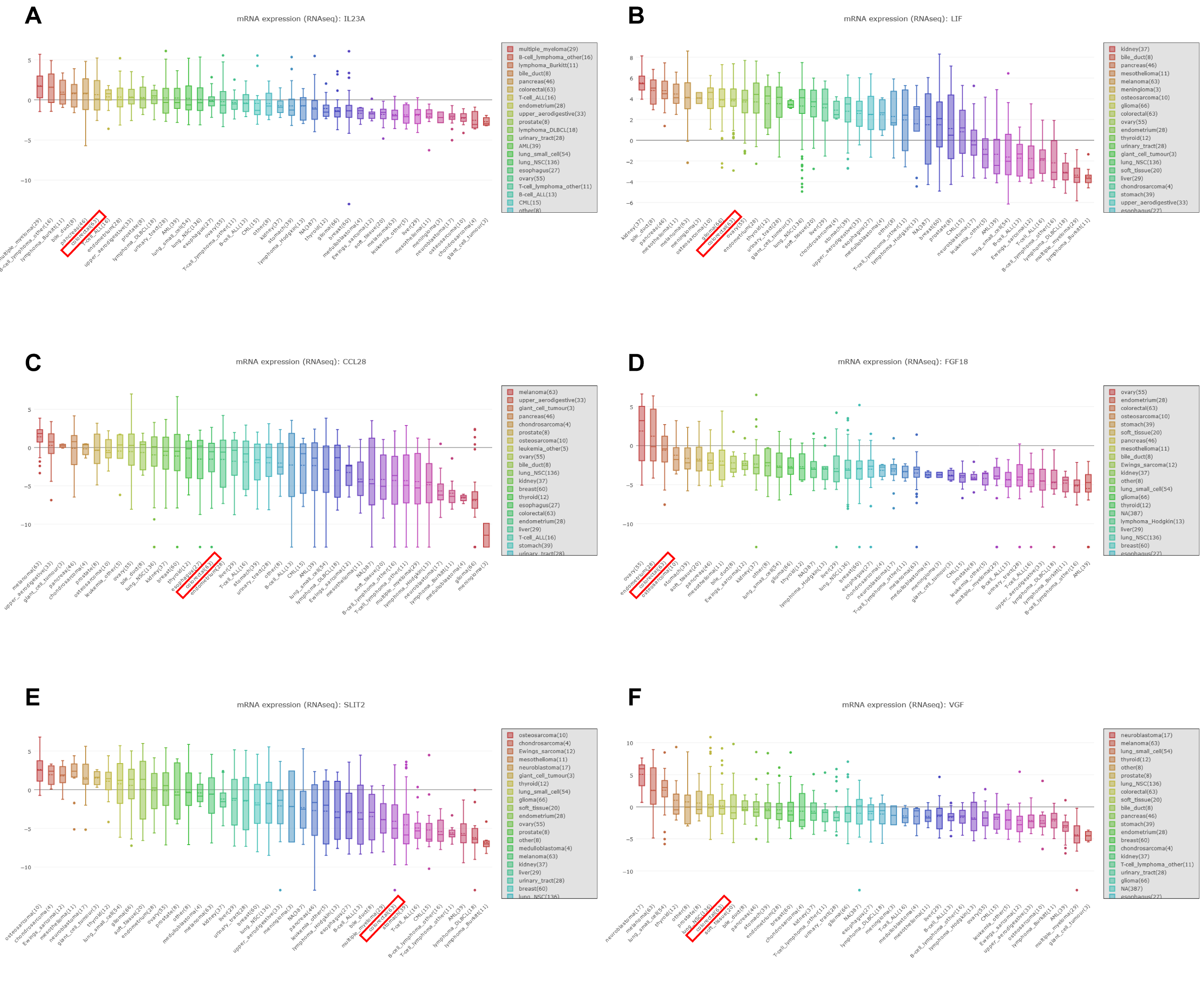
**Supplementary Figure 7**. The expression levels of *IL23A* **(A)**, *LIF* **(B)**, *CCL28* **(C)**, *FGF18* **(D)**, *SLIT2* **(E)**, and *VGF* **(F)** in various tumor cell lines in the Cancer Cell Line Encyclopedia (CCLE).


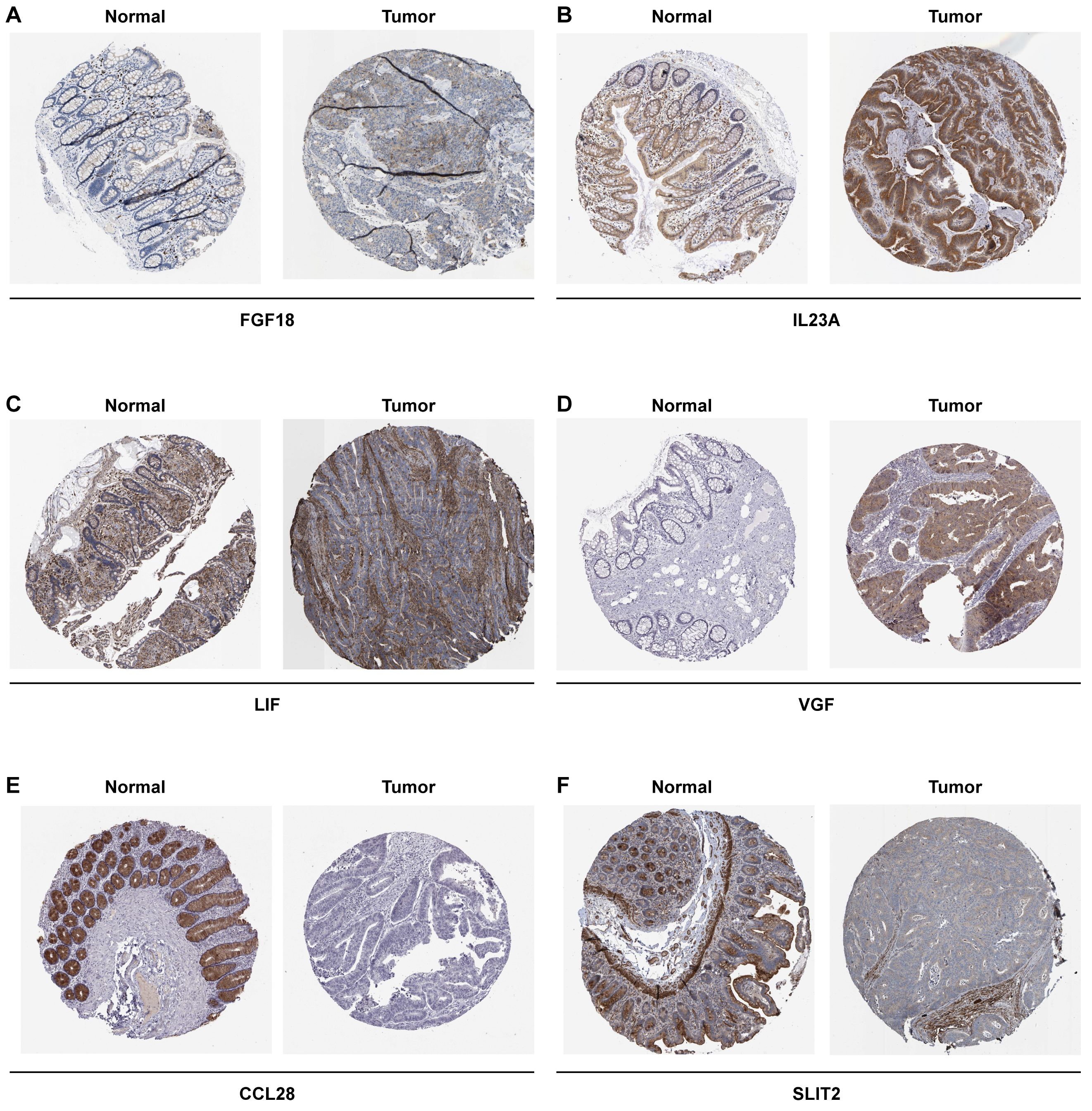


**Supplementary Figure 8**. Representative immunohistochemical results of FGF18 **(A)**, IL23A **(B)**, LIF **(C)**, VGF **(D)**, CCL28 **(E)**, and SLIT2 **(F)** in CRC and normal tissues from the [Human Protein Atlas](http://www.baidu.com/link?url=qFsa7cmVENqTJyMAFwRzlAcHk5LcvRpITqJkR3VwmQjUXMM2_pYaZ4ZSJ9s79l_B) (HPA) database. Abbreviation: CRC, colorectal cancer.

## Supplementary Tables

**Supplementary Table 1.** List of the top 20 upregulated and top 20 downregulated genes in differential immune-related gene analysis.

| **Symbol** | **Type** | **Log2FC** | ***p-*value** | **FDR** |
| --- | --- | --- | --- | --- |
| ESM1 | Up | 5.526410423 | 1.22E-257 | 3.62E-254 |
| CXCL5 | Up | 5.027090748 | 8.12E-49 | 1.42E-47 |
| INHBA | Up | 5.016970414 | 8.01E-156 | 4.40E-153 |
| IGF2 | Up | 4.674304115 | 5.64E-30 | 4.10E-29 |
| IL11 | Up | 4.65370376 | 3.71E-70 | 1.64E-68 |
| FABP6 | Up | 4.65088069 | 6.75E-88 | 6.07E-86 |
| MUC5AC | Up | 4.138133847 | 4.08E-27 | 2.59E-26 |
| TG | Up | 4.103383331 | 9.20E-70 | 4.01E-68 |
| S100A2 | Up | 4.096901702 | 2.78E-75 | 1.48E-73 |
| SPP1 | Up | 3.820434212 | 1.99E-41 | 2.44E-40 |
| STC2 | Up | 3.558137601 | 1.05E-71 | 4.90E-70 |
| OLR1 | Up | 3.47352059 | 2.32E-47 | 3.74E-46 |
| LCN15 | Up | 3.410632621 | 2.11E-14 | 6.71E-14 |
| DEFA6 | Up | 3.369249671 | 3.63E-16 | 1.27E-15 |
| CXCL8 | Up | 3.295205077 | 2.58E-35 | 2.42E-34 |
| TNFSF9 | Up | 3.254642242 | 2.25E-42 | 2.88E-41 |
| APLN | Up | 3.245495918 | 1.64E-92 | 1.85E-90 |
| AZGP1 | Up | 3.244055978 | 1.64E-71 | 7.61E-70 |
| BMP7 | Up | 3.206858661 | 1.73E-39 | 1.95E-38 |
| TDGF1 | Up | 3.020500994 | 1.25E-49 | 2.27E-48 |
| GUCA2A | Down | −5.052819021 | 1.02E-69 | 4.42E-68 |
| CHGA | Down | −4.478652101 | 3.81E-35 | 3.53E-34 |
| CHP2 | Down | −3.894110404 | 1.12E-48 | 1.93E-47 |
| GREM2 | Down | −3.761395943 | 1.29E-49 | 2.32E-48 |
| DES | Down | −3.739604273 | 4.15E-28 | 2.76E-27 |
| LIFR | Down | −3.502570916 | 3.14E-83 | 2.40E-81 |
| MASP1 | Down | −3.387743664 | 3.15E-33 | 2.66E-32 |
| VIP | Down | −3.341127304 | 1.23E-30 | 9.18E-30 |
| EDN3 | Down | −3.191214902 | 6.95E-44 | 9.56E-43 |
| FABP2 | Down | −3.147782162 | 3.45E-32 | 2.79E-31 |
| SEMA6D | Down | −3.035735771 | 1.03E-68 | 4.27E-67 |
| CR2 | Down | −2.917905961 | 2.68E-19 | 1.13E-18 |
| SECTM1 | Down | −2.876407377 | 7.24E-50 | 1.33E-48 |
| PTN | Down | −2.785680303 | 5.64E-68 | 2.26E-66 |
| IL6R | Down | −2.776907827 | 3.38E-105 | 5.69E-103 |
| NR3C2 | Down | −2.774275572 | 4.49E-71 | 2.05E-69 |
| CXCL12 | Down | −2.771946229 | 7.18E-63 | 2.30E-61 |
| TPM2 | Down | −2.678586817 | 5.49E-53 | 1.14E-51 |
| CD22 | Down | −2.603327995 | 1.93E-26 | 1.18E-25 |
| BMP5 | Down | −2.596512286 | 6.78E-28 | 4.46E-27 |

Abbreviation: FC, fold change; FDR, False discovery rate.

**Supplementary Table 2.** The results of stepwise multivariate Cox regression analysis.

| **Gene symbol** | **Coef** | **HR** | **HR.95L** | **HR.95H** | ***p*-value** |
| --- | --- | --- | --- | --- | --- |
| IL23A | 0.506 | 1.659 | 1.206 | 2.284 | 0.002 |
| LIF | 0.766 | 2.152 | 1.327 | 3.489 | 0.002 |
| VGF | 0.384 | 1.468 | 1.164 | 1.852 | 0.002 |
| SLIT2 | 0.179 | 1.196 | 0.980 | 1.461 | 0.079 |
| CCL28 | −0.190 | 0.827 | 0.690 | 0.991 | 0.039 |
| FGF18 | 0.350 | 1.420 | 0.996 | 2.025 | 0.053 |

Abbreviation: Coef, regression coefficient; HR, hazard ratio; HR.95L, 95% confidence interval; HR.95H. 95% confidence interval.

**Supplementary Table 3. |** List of differential expression transcription factor.

| **TF** | **logFC** | ***p-*value** | **FDR** |
| --- | --- | --- | --- |
| SALL4 | 4.955882576 | 1.84E-130 | 5.35E-128 |
| PDX1 | 4.703260455 | 5.27E-167 | 5.21E-164 |
| CBX2 | 3.657776326 | 9.00E-123 | 2.34E-120 |
| TFAP2A | 3.193415929 | 3.33E-40 | 3.86E-39 |
| TEAD4 | 2.350250173 | 1.45E-121 | 3.64E-119 |
| ARID3A | 2.261856067 | 1.37E-44 | 1.95E-43 |
| FOSL1 | 2.243754982 | 1.06E-29 | 7.61E-29 |
| TP73 | 2.200631436 | 2.77E-32 | 2.25E-31 |
| MYBL2 | 2.167067994 | 1.17E-62 | 3.69E-61 |
| LEF1 | 2.088066448 | 3.66E-41 | 4.41E-40 |
| RBP2 | 2.083611316 | 6.22E-12 | 1.72E-11 |
| TCF7 | 1.973203001 | 2.91E-67 | 1.15E-65 |
| MYC | 1.967588758 | 6.66E-72 | 3.13E-70 |
| SOX9 | 1.899854027 | 1.22E-78 | 7.50E-77 |
| E2F1 | 1.899271893 | 8.67E-66 | 3.14E-64 |
| E2F7 | 1.822533289 | 1.52E-58 | 4.00E-57 |
| CBX8 | 1.741574468 | 5.40E-113 | 1.10E-110 |
| SOX4 | 1.692377887 | 5.94E-72 | 2.81E-70 |
| CEBPB | 1.592977783 | 1.60E-39 | 1.81E-38 |
| FOXA2 | 1.454074792 | 7.75E-30 | 5.61E-29 |
| POLR3G | 1.435472349 | 5.84E-27 | 3.67E-26 |
| CENPA | 1.431627262 | 2.40E-54 | 5.30E-53 |
| FOXP3 | 1.399827642 | 2.06E-20 | 9.20E-20 |
| RUNX1 | 1.367713066 | 4.82E-53 | 1.01E-51 |
| FOXM1 | 1.348721976 | 1.69E-37 | 1.71E-36 |
| EZH2 | 1.268565703 | 2.34E-66 | 8.78E-65 |
| CBFB | 1.263736069 | 1.62E-68 | 6.69E-67 |
| BRCA1 | 1.182141291 | 1.18E-38 | 1.28E-37 |
| NCAPG | 1.18078315 | 2.81E-34 | 2.50E-33 |
| BHLHE40 | 1.161528371 | 8.77E-26 | 5.21E-25 |
| BATF | 1.145673265 | 7.64E-10 | 1.83E-09 |
| H2AFX | 1.125009797 | 1.70E-23 | 8.93E-23 |
| PRKDC | 1.124898783 | 4.62E-29 | 3.21E-28 |
| CBX3 | 1.062115713 | 8.02E-34 | 6.96E-33 |
| CHD7 | 1.054753991 | 6.63E-29 | 4.57E-28 |
| TRIM28 | 1.010066564 | 2.04E-29 | 1.44E-28 |
| SOX17 | −1.040139827 | 5.21E-12 | 1.45E-11 |
| VDR | −1.093713016 | 7.81E-35 | 7.14E-34 |
| TAL1 | −1.131571703 | 5.87E-15 | 1.92E-14 |
| EPAS1 | −1.218078714 | 1.45E-41 | 1.79E-40 |
| LMO2 | −1.244960401 | 8.24E-27 | 5.14E-26 |
| MITF | −1.324854999 | 2.57E-15 | 8.61E-15 |
| FLI1 | −1.335694786 | 6.65E-25 | 3.76E-24 |
| WWTR1 | −1.366593814 | 2.72E-18 | 1.08E-17 |
| PBX1 | −1.478575389 | 1.83E-29 | 1.30E-28 |
| HOXB13 | −1.480722161 | 1.83E-08 | 4.00E-08 |
| TCF7L1 | −1.553321507 | 3.52E-45 | 5.16E-44 |
| EBF1 | −1.577681426 | 1.00E-26 | 6.21E-26 |
| IKZF1 | −1.624479306 | 1.06E-22 | 5.39E-22 |
| RUNX1T1 | −1.69372425 | 2.58E-24 | 1.42E-23 |
| MXI1 | −1.701485802 | 1.85E-98 | 2.72E-96 |
| MEF2C | −1.704171352 | 1.99E-37 | 2.02E-36 |
| MAF | −1.76987740 | 6.64E-38 | 6.93E-37 |
| KAT2B | −1.834550761 | 4.50E-55 | 1.03E-53 |
| CBX7 | −1.855122214 | 1.25E-71 | 5.82E-70 |
| NR3C1 | −1.961975058 | 1.33E-42 | 1.72E-41 |
| AR | −1.98735271 | 1.43E-19 | 6.14E-19 |
| MEIS1 | −2.084912523 | 7.15E-38 | 7.44E-37 |
| TCF21 | −2.254054124 | 4.80E-52 | 9.65E-51 |
| NR5A2 | −2.293051691 | 1.72E-61 | 5.24E-60 |
| IRF4 | −2.459827805 | 1.99E-32 | 1.62E-31 |
| FOXP2 | −2.460956796 | 3.75E-25 | 2.16E-24 |
| KLF4 | −2.470752951 | 6.14E-66 | 2.24E-64 |
| MYH11 | −4.571879281 | 1.37E-56 | 3.38E-55 |
| SPIB | −4.701751068 | 4.36E-84 | 3.44E-82 |

Abbreviation: TF, transcription factor.

**Supplementary Table 4.** Preliminary clinical patient demographics and clinical characteristics.

| **Characteristic** | **Value** |
| --- | --- |
| Patients met criteria, n | 30 |
| Mean age, y | 58.200±15.568 |
| Gender, n |  |
| Male | 19(63.333) |
| Female | 11(36.667) |
| T stage, n |  |
| T3 | 29(96.667) |
| T4 | 1(3.333) |
| Chemotherapy, n |  |
| Yes | 7(23.333) |
| No | 23(76.667) |
| DFS event, n |  |
| Yes | 9(30.000) |
| No | 21(70.000) |

Data are expressed as n (%) or mean ± standard deviation. Abbreviation: y, years.
